# Robotic platforms in gynaecological surgery: past, present, and future

**DOI:** 10.52054/FVVO.16.2.024

**Published:** 2024-06-28

**Authors:** M Pavone, A Baroni, C Taliento, M Goglia, L Lecointre, A Rosati, A Forgione, Cherif Akladios, G Scambia, D Querleu, J Marescaux, B Seeliger1,2,7,9

**Affiliations:** IHU Strasbourg, Institute of Image-Guided Surgery, Strasbourg, France, 67091; IRCAD, Research Institute against Digestive Cancer, Strasbourg, France, 67091; Dipartimento di Scienze per la salute della Donna e del Bambino e di Sanità Pubblica, Fondazione Policlinico Universitario A. Gemelli, IRCCS, UOC Ginecologia Oncologica, Rome, Italy, 00168; Department of Obstetrics and Gynecology, University Hospital Ferrara, Ferrara, Italy, 44100; Department of Obstetrics and Gynecology, University Hospitals Leuven, Leuven 3000, Belgium, 3000; Department of Medical and Surgical Science Sciences and Translational Medicine, Faculty of Medicine and Psycology, Sapienza University of Rome, Rome Italy; ICube, UMR 7357 CNRS, University of Strasbourg, Strasbourg, France 67000; University Hospitals of Strasbourg, Department of Gynecologic Surgery, Strasbourg, France, 67091; University Hospitals of Strasbourg, Department of Digestive and Endocrine Surgery, Strasbourg, France, 67091

**Keywords:** Robotic-assisted surgery, minimally invasive surgery, multi-port, single-port, artificial intelligence, image-guided surgery

## Abstract

**Background:**

More than two decades ago, the advent of robotic laparoscopic surgery marked a significant milestone, featuring the introduction of the AESOP robotic endoscope control system and the ZEUS robotic surgery system. The latter, equipped with distinct arms for the laparoscope and surgical instruments, was designed to accommodate remote connections, enabling the practice of remote telesurgery as early as 2001. Subsequent technological progress has given rise to a range of options in today’s market, encompassing multi- port and single-port systems, both rigid and flexible, across various price points, with further growth anticipated.

**Objective:**

This article serves as an indispensable guide for gynaecological surgeons with an interest in embracing robotic surgery.

**Materials and Methods:**

Drawing insights from the experience of the Strasbourg training centre for minimally invasive surgery (IRCAD), this article offers a comprehensive overview of existing robotic platforms in the market, as well as those in development.

**Results:**

Robotic surgical systems not only streamline established operative methods but also broaden the scope of procedures, including intra- and transluminal surgeries. As integral components of the digital surgery ecosystem, these robotic systems actively contribute to the increasing integration and adoption of advanced technologies, such as artificial intelligence-based data analysis and support systems.

**Conclusion:**

Robotic surgery is increasingly being adopted in clinical practice. With the growing number of systems available on the marketplace, the primary challenge lies in identifying the optimal platform for each specific procedure and patient. The seamless integration of robotic systems with artificial intelligence, image- guided surgery, and telesurgery presents undeniable advantages, enhancing the precision and effectiveness of surgical interventions.

**What is new?:**

This article provides a guide to the robotic platforms available on the market and those in development for gynaecologists interested in robotic surgery.

## Introduction

Robotic surgery is developing together with new technologies gaining a prevalent role in surgical settings (Pavone et al., 2023a). Robot-assisted surgery (RAS) has become a surgical approach that has been increasingly used globally and the robotic device market is expected to raise its value by 15.9% over the next ten year. In past decades, one has witnessed a rapid shift from open to minimally invasive surgery, accompanied by the emergence of robotic surgery 25 years ago, first with a conventional approach and currently with the possibility of endoluminal procedures. However, notwithstanding the recognised benefits, the diffusion of robotic surgery has been slow. It is partly attributed to low reimbursements and high costs, which are only now gradually decreasing due to a growing competition from novel platforms in the marketplace. Gynaecologic surgery, which requires dealing with the upper abdomen (cytoreductive oncologic surgery and sometimes deep endometriosis) and the pelvis, has always benefited from the advantages of robotic surgical systems. Gynaecologic surgeons, when compared to general surgeons, only spend a portion of their training in the operating room, since they need to address several disciplines such as obstetrics, ultrasound, as well as dysfunctional endocrine pathologies. Minimally invasive surgery (MIS) calls for a lengthy learning curve, which decreases when transitioning to robotic surgery, facilitating a quicker shift from open surgery to MIS. Promoting robotic surgery in this speciality appears to be a winning strategy to expedite the MIS surgical transition.

The first robotic platforms designed date back to the 1980s-90s were predominantly utilised by orthopaedics (Robodoc Surgical System) and urology (Probot, Imperial College, London, United Kingdom) (Li and Chiu, 2018). Commercial advances introduced the Automated Endoscopic System for Optimal Positioning AESOP, (Computer Motion, California, United States) and a voice-controlled robotic endoscopic camera arm, which was also part of the table- mounted Zeus robotic system (Computer Motion, California, United States) (Marescaux et al., 2001). Following the merger between Computer Motion and Intuitive Surgical, the Zeus system was discontinued in favour of the da Vinci® surgical robotic system (Intuitive Surgical, California, United States), which for over more than two decades remains the most widely used robotic system in laparoscopic/thoracoscopic minimally invasive surgery (Lanfranco et al., 2004; Moran, 2006; Weinstein et al., 2009). To date, robotic approaches are used in approximately 2 to 15% of surgical procedures (Marcus et al., 2024). However, a variety of new robotic systems are commercially available in recent years, which is expected to lead to a further increase in adoption of robotic surgical approaches (Sheetz et al., 2020). Technological advances have led from multi-port systems (three or four separate arms) to single- port systems, where small-scale instruments are inserted through a single incision in the body. Recent developments include multi-articulated and flexible robotic devices that can operate on patients within their body lumens (Seeliger et al., 2022). As a result, not only has robotic surgery found a particular role in gynaecology, especially in obese patients with early endometrial carcinoma, where MIS is the gold standard, but also in the management of complex cases in endometriosis or urogynaecologic surgery (Pavone et al., 2023b; Pavone et al., 2023c; Restaino et al., 2020). With the swift technological evolution and the robust evidence supporting the advantages of MIS over conventional laparotomy, attention has shifted to exploring the additional benefits offered by these newly introduced robotic systems. In addition, given the growing array of available platforms, it is mandatory to clearly define the potential benefits and limitations of various systems. It is essential not only to select the best platform among available options for the single surgeon, but also to identify the optimal system tailored to the specific needs of individual patients or procedures. Robotic systems are then suitable for educational purposes, offering simulators that can democratise training for non- expert surgeons (Simmonds et al., 2021).

From the expertise gained at the IRCAD research and training centre in Strasbourg (France) where theoretical and hands-on robotic courses take place throughout surgical disciplines in collaboration with seven robotic industrial partners (Intuitive Surgical, Medtronic, CMR, EndoQuest, Momentis Surgical, Medicaroid, and Distalmotion), we aimed to provide an overview of the platforms currently commercialised in gynaecologic surgery along with other platforms that are emerging in the field.

## Robotic applications in gynaecology

Although the benefits of robotic surgery have been demonstrated in terms of surgical precision and ergonomics and non-inferiority in feasibility and safety has been reported, there are no published trials assessing the superiority of robotic surgery over laparoscopy in any gynaecologic condition. The main limitation to its dissemination is related to the platform and procedural costs, still not fully reimbursed by national health systems (D’Hondt et al., 2023). The benefits of robotic surgery over open surgery and conventional laparoscopy in gynaecology cover various aspects. In comparison to open surgery, robot-assisted procedures lead to reduced recovery times, shorter hospital stays, minimal scarring, and a notable reduction in blood loss, transfusions, and overall pain and discomfort. In patients with a high BMI, a lower number of complications has been observed (Sheetz et al., 2020).

As compared to conventional laparoscopy, robotic surgery offers superior dexterity with the robotic instruments outperforming the human hand in terms of range of motion and precision (Sheetz et al., 2020). The endo-wrist movement of the robotic instruments allows for improved precision in suture-intensive operations, helping surgeons to perform tasks that would be challenging without any robotic assistance (Pavone et al., 2023b). Robot-assisted surgery reduces surgical tiredness and muscular strain, particularly during extended or multiple surgeries in a single day, promoting precision and potentially reducing the frequency of medical errors (Haffar et al., 2023). Robotic surgery also provides better access to difficult- to-reach locations due to increased flexibility and precision. The surgeon, seated in an ergonomically comfortable posture at the console, has superior surgical autonomy, controlling the camera and operative arms. The technology offers a better visual field, allowing for more precise surgery and improving outcomes in conditions such as endometriosis and cancer (Green et al., 2023). The learning curve for robotic surgery is shorter as compared to conventional laparoscopic surgery, leading to fewer open surgery conversions (Saqib et al., 2023). Blood loss and transfusions are further reduced, and there is less pain and discomfort, thanks to the dexterity of the robotic tool tips, minimising excessive leverage and force at incision sites. The benefits extend towards a shorter hospital stay, reduced recovery time, and fewer problems overall, except for surgeons with limited experience. Despite these benefits, it is crucial to acknowledge some disadvantages of robotic surgery, including high initial installation and maintenance costs, potential movement latency during emergencies, longer operating times in the early learning stages, and the need for additional personnel and training (Gueli Alletti et al., 2022). However, with an experienced team, such challenges can be mitigated, and the benefits of robotic surgery in gynaecology remain significant (Restaino et al., 2020). When considering specific indications even if laparoscopic surgery is the gold standard in the treatment of endometriosis (Ianieri et al., 2023) when dealing with deep endometriosis cases, conventional surgery becomes complex and often requires a multidisciplinary surgical team. In such scenarios, RAS provides technical benefits including 3D visualisation, tremor filtering, and improved surgical manoeuvrability (Seeliger et al., 2019). A meta-analysis compared laparoscopic and RAS in endometriosis confirming that robotic surgery is a safe and feasible option, particularly when infiltrating lesions involve the bowel and the ureters (Restaino et al., 2020). As for endometriosis, robotic surgery is frequently adopted in the urogynaecologic field. In 2023, the first case series of robotic sacral colpopexies (RSCPs) using the HUGO™ RAS robotic system was reported (Panico et al., 2023), suggesting its effectiveness in both objective and subjective measures, with minimal intraoperative and postoperative complications (Khashab and Kalloo, 2011). Robotic approaches are also used as an alternative to laparoscopy in other simple procedures such as hysterectomies, myomectomies, and transabdominal cerclages (Hamza et al., 2023). To date, in gynaecologic oncology, robotic surgery has played a role in the treatment of obese early-stage endometrial cancer (EC) patients to overcome the issue of trocar positioning and the surgeon’s arm movements in larger abdomens (Corrado et al., 2015; Ran et al., 2014; Seror et al., 2014). A prospective randomised trial (ROBESE trial) comparing laparoscopy versus robotic surgery in such patients is underway to evaluate the conversion rate. In ovarian cancer (OC), MIS may only be the chosen approach in the definition of disease extension (the Fagotti score) (Marchetti et al., 2021), early-stage cases, selected cases of interval debulking surgeries (LANCE trial) (Nitecki et al., 2020) or specific recurrences (Fanfani et al., 2016b). In cervical cancer (CC), in 2018 the LACC trial (Ramirez et al., 2018) excluded the possibility of laparoscopy in patients with a tumour size >2cm suggesting that laparoscopic radical hysterectomy may have lower disease-free survival and overall survival rates as compared to open abdominal radical hysterectomy (Saqib et al., 2023). However, two prospective randomised clinical trials are underway to assess the outcomes and survival of CC patients undergoing robotic radical hysterectomy (RACC and ROCC trial) (Falconer et al., 2019; Bixel et al., 2022). Staging lymph nodes using the sentinel lymph node (SLN) technique is a pivotal procedure in the early stages of both endometrial and cervical carcinoma. Robotic platforms offer enhanced visualisation of images and facilitate the identification of SLN.

**Table I:** Ongoing clinical trials in robotic gynaecologic surgery.

Registry number	Sample	Country	Study phase	Condition/Disease	Intervention	Primary endpoint	Recruitment state
NCT05974995	566	Italy	Not applicable	Endometrial cancer, obesity	Robot-assisted surgery, laparoscopic surgery	Conversion rate	Not yet recruiting
NCT05362838	50	Austria	Not applicable	Endometriosis, endometriosis-related pain	Robot-assisted laparoscopy, conventional laparoscopy	Change in Visual analogue scale (VAS) from baseline to 3 and 6 months after the operation; Pain medication; Menstrual bleeding; Patients' Global Impression of Change (PGIC)	Recruiting
NCT03719547	800	Sweden	Not applicable	Cercival cancer	Abdominal radical hysterectomy, robot-assisted radical hysterectomy, sentinel lymph node biopsy	Recurrence-free survival	Recruiting
NCT05179109	70	Finland	Not applicable	Deep endometriosis	Minimally invasive surgery	NRS (Numeric rating scale) score for pain	Recruiting
NCT05357924	104	Austria	Not applicable	Ovarian endometrioma, ovarian endometriosis	Robot-assisted laparoscopy, conventional laporoscopy	Change in serum AMH (sAMH) from baseline to 6 months after the operation	Recruiting
NCT03633786	40	Italy	Not applicable	Endometriosis	Standard laparoscopic surgery, robot-assisted surgery, assessment of sexual function, assessment of bowel symptoms, assessment of urinary symptoms	Operating time	Active, not recruiting
NCT06050161	50	USA	Not applicable	Pelvic organ prolapse, abnormal uterine bleeding mesh, augmentation laparoscopic surgery, robotic surgical suturing, laparoscopic hysterectomy, minimally invasive surgery	Articulating laparoscopic instrument, conventional laparoscopic instrument, robotic surgical instrument	Mesh suturing time; Vaginal cuff suturing time	Not yet recruiting

However, of note, not all platforms are equipped with cameras that can detect the indocyanine green (ICG) tracer which is commonly used in this process (Table II). Ongoing prospective clinical trials are listed in Table III. Future research will focus on the discovery and applications of robotic platforms that go beyond the simple surgical approach. The benefits of such systems are likely to easily integrate robotic interfaces with artificial intelligence (AI) algorithms, augmented reality (AR) models, and image-guided surgical approaches (Pavone et al., 2024b; Pavone et al., 2023b).

**Table II:** Multi-port systems for gynaecologic surgery overview.

Robot	Field	Console	Ergonomics	Surgeon sterile	Vision	Long distance control	Bedside system	Instrument arms	Instruments	Trocar position	Trocars	Availability	Year
da Vinci® Xi (Intuitive)	GYN, ENT, GS, THOR, UR	CLOSED	SEATED	NO	3D HD + NIR	Yes	Single cart	3	REUSABLE	SPECIFIC	SPECIFIC	COMMERCIAL	2014 (FDA)
Sina robotic system (Sina Robotics and Medical Innovators)	GYN, GS	OPEN	SEATED + STANDING	NO	4K	Yes	Independent BSU	2	-	-	-	COMMERCIAL	2015 (IRAN)
Senhance (Asensus)	GYN, GS, THOR, UR	OPEN	SEATED	NO	3D HD + NIR	-	Independent BSU	2	REUSABLE	SPECIFIC	GENERIC	COMMERCIAL	2017 (FDA)
Revo-I (Revo)	GYN, UR	CLOSED	SEATED	NO	3D HD	-	Single cart	3	-	-	-	COMMERCIAL	2017 (KOREA)
Versius (CMR)	GYN, ENT, GS, THOR, UR	OPEN	SEATED + STANDING	NO	3D HD	Yes	Independent BSU	3	Country-specific	SPECIFIC	GENERIC	COMMERCIAL	2019 (FDA)
Avatera (avateramedical)	GYN, GS, UR	OPEN	SEATED	NO	3D HD	-	Single cart	3	DISPOSABLE	SPECIFIC	GENERIC	COMMERCIAL	2019 (CE)
Hinotori (Medicaroid)	GYN, UR	CLOSED	SEATED	NO	3D	Yes	Single cart	3	-	SPECIFIC	-	COMMERCIAL	2020 (Japan)
Dexter (Distalmotion)	GYN	OPEN	SEATED + STANDING	YES	-	-	Independent BSU	2	REUSABLE	AS IN MIS	GENERIC	COMMERCIAL	2020 (CE)
HUGO™ RAS (Medtronic)	GYN, UR	OPEN	SEATED	NO	3D HD	-	Independent BSU	3	-	SPECIFIC	GENERIC	COMMERCIAL	2021 (CE)
SSI (Mantra Innovation)	GYN, CARDIAC, ENT, GS, THOR, UR	OPEN	-	NO	3D HD	-	Independent BSU	4	-	-	-	COMMERCIAL	2022 (India)
Maestro (Moon Surgical)	GYN, GS, UR	Non-Console System	-	YES	3D	-	Single cart	2	-	SPECIFIC	GENERIC	COMMERCIAL	2022 (FDA)
Carina RAS (Ronovo Surgical)	GYN, UR	CLOSED	SEATED	NO	3D HD + NIR	-	Independent BSU	3	-	-	SPECIFIC	PROTOTYPE	-
Bitrack System (Rob Surgical)	GYN, GS, THOR, UR	OPEN	SEATED	NO	3D	-	Single cart	3	DISPOSABLE	AS IN MIS	GENERIC	PROTOTYPE	2018
Ottava (Ethicon/J&J)	GYN, GS, THOR, UR	-	SEATED	NO	3D HD + NIR		Independent table-mounted	5/6	-	-	-	PROTOTYPE	2024
Luna (Asensus)	GYN, GS, UR	OPEN	SEATED	NO	3D HD		Independent BSU	3	-	-	-	PROTOTYPE	2025

BSU: bedside units; NIR: Near-infrared fluorescence; GYN: Gynaecology; GS: General surgery; THOR: Thoracic surgery; ENT: head and neck surgery; CARDIAC: cardiac surgery; UR: urology

**Table III:** Single-port systems for gynaecologic surgery overview

ROBOT	FIELD	CONSOLE	ERGONOMICS	VISION	ACCESS	APPROACH	AVAILABILITY	YEAR
da Vinci® SP (Intuitive Surgical)	GYN, ENT, UR	CLOSED	SEATED	12x10mm articulating 3D Camera	RIGID 25mm	Laparoscopic	COMMERCIAL (ENT, UR) CLINICAL TRIAL (GYN)	2014 (FDA)
Flex® robotic system (Medrobotics)	GYN, ENT, GS	OPEN	SEATED + STANDING	3D HD	FLEX	Endoluminal/NOTES	COMMERCIAL	2015 (FDA)
Anovo (Momentis Surgical)	GYN	OPEN	SEATED	3D	RIGID	NOTES	COMMERCIAL	2021 (FDA)
Endoluminal Surgical System (ENDOQUEST Robotics)	GYN, ENT, GS	OPEN	SEATED	3.7mm HD	FLEX	Endoluminal/NOTES/Laparoscopic	CLINICAL TRIALS	2021
Enos (Titan Medical)	GYN, GS	OPEN	SEATED	3D HD	RIGID	Laparoscopic	PROTOTYPE	2020
Virtuoso (Virtuoso surgical)	GYN, BR, UR	OPEN	-	-	RIGID	Endoluminal	PROTOTYPE	2024
SPS (Colubris MX)	-	OPEN	SEATED	3D	RIGID	Laparoscopic	PROTOTYPE	-

GYN: Gynaecology; GS: General surgery; ENT: head and neck surgery; UR: urology; BR: bronchoscopy.

## Multi-Port Systems For Gynaecologic Procedures

The da Vinci® robotic surgical system manufactured by Intuitive Surgical, United States, has been a pioneering multi-port platform in robotic surgery over the past two decades, recognised as the primary player in the marketplace (Moran, 2006). In 1999, the first da Vinci® robotic platform introduced the four arms with simple surgical instruments. In 2006, with the da Vinci S®, we saw the introduction of the first 3D HD (720p) vision, and in 2014, the da Vinci Xi®, ready for future technologies, switched to crystal clear vision and multi-quadrant access. Robotic systems designed for laparoscopic surgery have classically comprised multi-port set-ups featuring a single camera arm and two or more instrument arms. In the conventional master-slave configuration, a surgeon console connects to either a multi-arm boom or multiple independent bedside units. Control is facilitated through hand- and foot-operated switches on the master console. These robotic arms are equipped with specialised articulating instruments that replicate human wrist movements, surpassing the range of motion exhibited via rigid laparoscopic instruments and even exceeding that of human wrists through clutching (>360-degree rotations). In the past decade, various systems with similar architecture and others with independent bedside units have reached the commercialisation stage. Most current robotic platforms are versatile and approved for various disciplines, notably general surgery, urology, and gynaecology. Three- dimensional (3D) vision, available in both closed- console systems with deep immersion and open- console systems in the operating room environment with flat screens, provides a realistic perception of anatomical structures. The latest generation devices offer high connectivity through wired or wireless connections. Independent bedside units, as exemplified by the Senhance® surgical system (Asensus Surgical, United States) with haptic feedback and non-articulating instrumentation (Fanfani et al., 2016a), the Versius Surgical System (CMR Surgical, United Kingdom) and the HUGO™ RAS System (Medtronic, Ireland) with articulating instrumentation but without haptic feedback, may be positioned around the operating table independently of one another (Soumpasis et al., 2023). The Medtronic platform was originally approved in Europe only for gynaecologic procedures with the first reports in 2022 for prophylactic hysterectomy, urogynaecology, and endometriosis treatment (Gueli Alletti et al., 2022; Panico et al., 2023; Pavone et al., 2023a). Concerns about the docking wasted time in robotic platforms and the impact on the total operating time and therefore on costs may be overcome. Even in platforms with independent bedside units, mean docking time appears to be cost-effective at 5.08 minutes (range: 2-12) (Howell et al., 2014). In a dual-console arrangement, instruments are operable from each surgeon console, promoting collaborative efforts between experienced surgeons and trainees. Surgeon consoles incorporate safety mechanisms to automatically lock robotic instruments when console surgeons divert their attention, such as by moving their heads or hands away from the controls. It is achieved through features such as head sensors, gaze direction detection on dedicated glasses, or hand contact sensors in the controllers.

Last-generation consoles offer alternatives such as the so-called on-demand robotic systems, focusing on a swift transition between laparoscopic and robotic approaches while keeping surgeons in a sterile environment. Examples include the Dexter system (Distalmotion, Switzerland) or the non-console system also known as Maestro (Moon Surgical, France) (Hamza et al., 2023). The upcoming OTTAVA robotic platform featured by Johnson & Johnson (FDA prototype submission in 2024 II trimester) is going to be the first system fully integrated into the “robotic” OR in a twin of motion with communication between the console and the patient’s bed.

The requisite range of tools depends on the surgical speciality, with interchangeable instruments encompassing basic electrosurgical equipment for most platforms and advanced vessel- sealing or stapling devices in later developmental stages. For a comprehensive overview of commercially available multi-port systems, including some advanced prototypes in preclinical or clinical trials, please refer to Table II.

In the foreseeable future, the advancement of remote RAS will contribute significantly to the democratisation of surgery. This innovative approach involves skilled surgeons manipulating robotic systems from distant locations, thereby expanding access to quality care. For instance, an experienced surgeon situated in an urban area could conduct robot-assisted surgery on a patient residing in a remote location lacking specialised medical centres. To realise this vision, essential technical components such as robust data transfer technology and a reliable network infrastructure must be established to prevent any surgical delays. Additionally, the creation of comprehensive telemedicine systems is imperative to facilitate seamless remote surgical interventions. Hinotori surgical system (Medicaroid, Japan) has spearheaded the development of guidelines for remote-assisted robotic surgery, providing essential support for the advancement of surgical practices in this domain.

## Single-Port Systems For Gynaecologic Procedures

Minimising the number of ports and incisions while maintaining optimal manoeuvrability and effectiveness poses a challenge for MIS. A review of the literature including 1,065 patients was performed pertaining to robotic single- site surgery (RSSS) in gynaecology. There, the authors reported no significant differences in terms of operating time, estimated blood loss, and hospital stay with multi-port approaches suggesting that RSSS is viable for a wide range of gynaecologic surgical interventions irrespective of benign/malignant conditions (Capozzi et al., 2021). Single-port laparoscopic procedures elicit greater technical tasks as compared to multi-port approaches due to the proximity of instruments and of the camera, resulting in reduced triangulation and potential collisions. Single-port platforms initially originated from the adaptation of a multi- port system with the da Vinci® SP system (first FDA-approved in 2014) usable in gynaecologic procedures only in clinical trials but approved for transoral and urologic surgeries. The increased learning curve and the restricted ergonomics together with the increased risk of incisional hernias when compared to 10mm ports and the impossibility of having suction or additional instruments limited the spread of these systems. However, nowadays, the progression of single-port robotic platforms, both rigid and flexible, opens up avenues to explore new anatomical targets within the respiratory, gastrointestinal, and genitourinary systems with endoluminal and transluminal approaches (Mascagni et al., 2019; Seeliger and Swanström, 2020). Presently, the MIRA platform (from Virtual Incision, United States, weighing 1.5kg) boasts the smallest rigid access point at 15mm, and it does not necessitate any dedicated operating room (Seeliger et al., 2019; Marks et al., 2021; Kim et al., 2023). Foetal medicine is a domain in which single-port transvaginal surgery is explored, with prototypes such as Colubris MX currently in development (Seeliger et al., 2022). The Flex® robotic system manufactured by Medrobotics has found applications such as a flexible platform in gynaecology (Seeliger and Swanström, 2020). The Anovo system manufactured by Momentis Surgical represents the latest addition to the marketplace; it is designed specifically for transvaginal benign gynaecologic indications. This system achieves a “laparoscopic” intra-abdominal instrument setting, even with a V-NOTES approach, due to its complete instrument flexibility (Allemann et al., 2010) (Khashab and Kalloo, 2011). EndoQuest Robotics is available for clinical studies in gynaecology, gastroenterology, and general surgery (Kim et al., 2023).

## Upcoming Perspectives

The progression of surgery has led from a relatively safe but often ineffective ‘simple’ approach to complex, effective, yet potentially risky procedures (Howell et al., 2014). Now, the challenge is to train the surgeon and to develop the operating room of the future. In the era of digital surgery, robotic platforms represent computer interfaces which can integrate multiple modalities of real- time data analysis). In this way, advanced systems can offer augmented surgical vision through AR, improved surgical decisions with AI, and enhanced surgical manoeuvres with the evolution of robotic instruments. The incorporation of preoperative planning through 3D acquisition of radiological images, coupled with the creation of non-rigid AR models that synchronise with the patient’s natural respiratory and surgical movements and the use of deep learning (DL) algorithms to analyse surgical phases, constitutes an ideal toolkit to enhance robotic surgery (Pavone et al., 2024b). This comprehensive approach aims to reduce the incidence of intraoperative complications and optimise surgical outcomes limiting surgical discrepancies. The operating room will increasingly be a control centre such as an airport control tower, which can handle 2D/3D inputs derived from preoperative images, environmental and laparoscopic cameras, and patient physiological signals, and returning outputs to the robotic platforms offering information on the surgeon’s screen for intraoperative processes (e.g., remaining operating time or patient clinical situation). Image-guided surgery and in particular intraoperative ultrasound is gaining a role in robotic surgery (Mascagni and Padoy, 2021). Drop-in ultrasound probes can be easily handled by means of the robotic grasper, reaching difficult anatomical spaces (Pavone et al., 2024b; Guerra et al., 2015). Intraoperative ultrasound with images that can be projected onto the surgeon’s screen thanks to the platform offered by Intuitive Surgical (TilePro) may help with surgical radicality in endometriosis and with the distinction between healthy and diseased tissue in oncology (Giannone et al., 2021).

## Conclusion

In conclusion, we are at the dawn of a transformative era in surgery. The increasing accessibility of robotic platforms, coupled with cost reductions, is heralding a paradigm shift. While prospective trials have yet to definitively establish superiority, the evident benefits are reshaping the surgical landscape. The seamless integration of these platforms with artificial intelligence (AI) and augmented reality (AR) systems aligns with the vision of a fully integrated operating room. The emergence of novel platforms using endoluminal and flexible approaches is revolutionising the very essence of surgical practice. This evolution promises not only increased minimisation of invasiveness but also yields superior cosmetic outcomes. As we progress in this evolving surgical landscape, the potential for further innovation and refinement underscores the dawn of a new era, where surgery embraces unprecedented advances for the sake of both surgeons and patients alike.
